# STAYING GOLDEN: PERSPECTIVES FROM THE AGE 85+ ON THE REALITIES OF AGEISM

**DOI:** 10.1093/geroni/igae098.0094

**Published:** 2024-12-31

**Authors:** Taylor Patskanick, Sophia Ashebir, Lisa D’Ambrosio, Joseph Coughlin

**Affiliations:** Massachusetts Institute of Technology, Cambridge, Massachusetts, United States; Massachusetts Institute of Technology, Cambridge, Massachusetts, United States; MIT AgeLab, Cambridge, Massachusetts, United States; MIT AgeLab, Cambridge, Massachusetts, United States

## Abstract

The conceptualization, prevalence, and consequences of ageism are increasingly well-documented in research, but our understanding of how older people may personally experience this phenomenon during specific age periods in their older adulthood is less understood (e.g., Chasteen et al., 2021). The 85+ age cohort may experience unique forms of ageism and age-based discrimination relative to other older age groups and can comment on how they experience these biases relative to other ages in their own lifespan. This paper shares findings from a qualitative, cross-sectional study with the MIT AgeLab 85+ Lifestyle Leaders panel, a longitudinal panel of U.S. octogenarians and nonagenarians. Utilizing four virtual focus groups (N=17) conducted in January 2024, findings were analyzed in MAXQDA2024 using a team-based, inductive, thematic analysis approach. Results characterize Lifestyle Leaders’ experiences with felt forms of ageism (e.g., covert vs overt, positive vs negative, etc). The Lifestyle Leaders articulated experiences with ageism in specific domains such as product design, healthcare, and in the community-based built environment. Benevolent or compassionate ageism may be particularly amplified at age 85+. Lifestyle Leaders noted an inextricable intersectionality between their chronological age and social identities like physical ability, digital nativity, and gender. A plurality described having a longitudinal lens on the shifting institutional context for age-based discrimination over their life courses (e.g., ADEA). These findings and their implications for gerontological professionals and future research on ageism, including planned follow up to this study examining this topic via participatory action research methods, will be discussed.

